# Climate and Hydrology Shape the Growth and Water Use Efficiency in South Florida's (USA) Pine and Cypress Forests

**DOI:** 10.1002/ece3.73253

**Published:** 2026-04-05

**Authors:** Manuel Bernal‐Escobar, Courtney L. Angelo, Kenneth J. Feeley

**Affiliations:** ^1^ Department of Biology University of Miami Coral Gables Florida USA; ^2^ National Park Service Big Cypress National Preserve Ochopee Florida USA; ^3^ Fairchild Tropical Botanic Garden Coral Gables Florida USA

**Keywords:** climate change, dendroecology, intrinsic water use efficiency, seasonal flooded forest, tree growth rates

## Abstract

In south Florida, climatic variation and ongoing hydrologic alterations are predicted to impact the growth and ecophysiological performance of tree species. Understanding how tree growth rates vary across temporal and landscape‐scales in relation to climate and hydrology will help us better understand and predict how tree species, and thus landscapes will respond to increasing variability in regional climates. Here, we used dendrochronology and stable isotopes (δ^13^C) to examine tree growth rates and intrinsic water‐use efficiencies (iWUE) of *Taxodium ascendens* , *T. distichum* , and *Pinus elliottii* in relation to climate and hydrology at the southern ends of their distributions. Specifically, we cored 20–26 individuals of each tree species growing in south Florida's Big Cypress National Preserve to estimate annual growth rates and iWUE. From these samples, we built tree‐ring chronologies and determined δ^13^C. Then we evaluated how both growth rates and iWUE vary with climatic and hydrological conditions through time and across the landscape. Although overall climate–growth correlations were weak, water depth proved influential. *Taxodium ascendens* and *T. distichum* grew most rapidly during summers (June–September) when seasonal standing water depths increase. In contrast, *P. elliottii* grew faster in springs (April–May) when seasonal standing water depths are the lowest. Relative location within the landscape was not an important factor driving tree growth. The iWUE of all species increased significantly with rising mean annual temperature and vapor pressure deficit (VPD), while precipitation and water depth had differential effects on each species' iWUE. Overall, our results highlight the complexity of factors driving tree growth rates and iWUE of these tree species at the southern ends of their distributions, as well as the potential for future climate‐driven changes in tree growth and performance across south Florida's natural ecosystems.

## Graphical abstract

In South Florida, tree growth and water‐use efficiency are significantly influenced by hydrological conditions, with water depth playing a more critical role than overall climate variables. Using dendrochronology and stable carbon isotopes, the study found that *Taxodium* species grow best during wetter summers, while *Pinus elliottii* grows most in wetter springs, with intrinsic water‐use efficiency increasing alongside temperature, vapor pressure deficit, and atmospheric carbon concentration. These findings highlight the complex interactions between hydrology, climate, and tree physiology, suggesting that future changes in water availability could significantly impact forest dynamics in the region.

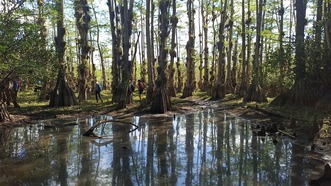

## 1 Introduction

South Florida's natural landscape is a mosaic of wetland and upland ecosystems dominated by a small number of tree species (Gunderson and Loope 1982; Schipper 2022). Among these, two cypress species *Taxodium ascendens* Brongn. (pond cypress, incl. “dwarf” cypress) and *T. distichum* (L.) Rich. (bald cypress) and slash pine ( *Pinus elliottii* Engelm., = *Pinus elliottii var. densa* (Little & Dor.)) are particularly important due to their abundance, dominance, expansive distributions, and influences on ecosystem structure and function.

These three tree species inhabit diverse hydrological settings ranging from year‐round flooded wetlands to drier pine flatwoods, making them ideal indicators of how trees respond to environmental variability and climate change in subtropical south Florida (Muss et al. 2003). *Taxodium distichum* establishes around cypress “domes” depressions with deep water and year‐round inundation (i.e., 250–365 days); *T. ascendens* thrive in cypress prairies and in ecotones between the prairies and cypress domes with marked hydroperiod fluctuations (i.e., prairies are flooded up to 120 days and ecotones between 200 and 250 days); and *P. elliottii* is drought‐adapted and grows in pine uplands that flood only occasionally (i.e., 20–60 days) (Duever et al. 1979). While all three tree species vary in hydroperiods, the depth of water during each hydroperiod also varies based on elevations associated with each plant community (Duever et al. 1979). Additionally, all three species are temperate‐affiliated and reach the southern/hotter range of their distributions in southern Florida, making them ideal focal species to study how climate change, increasing temperatures, and hydrologic variability affect tree performance at species' trailing geographic edges.

In south Florida, mean annual temperatures have been increasing by approximately 0.025°C per year since the late 1970s (PRISM Climate Group and Oregon State University 2023; Climate Change Response Program 2024). In addition to rapidly rising temperatures, there have been changes in the timing and magnitude of precipitation events, with general increases in atmospheric vapor pressure deficit (VPD) (IPCC 2021; PRISM Climate Group and Oregon State University 2023). Seasonal rainfall patterns from May to October (wet season) and from November to April (dry season) (Meteoblue 2025; PRISM Climate Group and Oregon State University 2023) govern regional hydroperiods, with prolonged inundation during wetter months and reduced water availability in the dry season. Future climate projections for south Florida include regional temperature increases between 2.2°C and 4.4°C by 2100. In addition, more intense wet‐season downpours, longer dry‐season droughts, and sea‐level rise are all predicted to further impact the hydroperiod in south Florida (Carter et al. 2014).

Since the early 1900s, the creation of extensive canal networks around Lake Okeechobee has dramatically altered natural water flows and levels in south Florida (Duever et al. 1979; Dunn 1967). At the same time, the south Florida landscape experiences regular disturbances such as fires, floods, hurricanes, and was subject to selective logging at the end of the nineteenth century. The fire, floods, and hurricane frequencies and intensities are linked to weather seasonality and variability. For example, floods are a consequence of increased precipitation during the wet season, while fires are common in the dry season. However, in dry years, less area is inundated by standing water, which allows for larger and more intense fires (Duever et al. 1979; Dunn 1967). Together, these concurrent shifts in climate, hydrology, and land use can all critically influence tree growth rates and ecosystem dynamics in south Florida. In addition, increasing atmospheric CO_2_ concentrations (*C*_a_) can also change plant water use (Wang et al. 2024), the carbon fertilization effect (Keenan et al. 2013), and thereby affect plant performance and ecosystem dynamics. However, the combined impacts of rising *C*_a_, climate change, and water management on the dynamics of south Florida's natural forested ecosystems remain poorly understood.

Tree rings and stable isotopes have played a very important role in characterizing patterns of tree growth in many different tree species and systems (Andreu‐Hayles et al. 2023; Zhao et al. 2019; Zuidema et al. 2022), but have only rarely been used to understand tree dynamics in subtropical south Florida. This is in part because these systems are less seasonal, which may prevent the formation of distinct and reliable annual growth rings (Tomlinson and Craighead 1972). However, in the last 20 years tropical and subtropical dendrochronology has flourished, and more than 347 species have been used in tree‐ring research worldwide with different applications in ecology, climatology, and other disciplines (Quesada‐Román et al. 2022; Andreu‐Hayles et al. 2023). Indeed, previous studies have demonstrated that *T. ascendens, T*. *distichum*, and *P. elliottii* trees in south Florida can in fact all produce reliable growth rings (Anderson et al. 2005; Harley et al. 2012). These annual growth rings can allow for the construction of tree‐ring chronologies as well as accurate determinations of tree ages and growth rates based on longer temporal scales. These data can in turn be used to assess the effects of environmental and climatic pressures on tree growth (Zampieri et al. 2024; Bernal‐Escobar et al. 2025). Trees growing in seasonally flooded forests have also been shown to be more sensitive to water‐related climatic variables such as precipitation, water depth, and VPD than trees in non‐flooded systems (Schöngart et al. 2002, 2004; López et al. 2014; Herrera‐Ramirez et al. 2017).

In addition to providing information on growth patterns, tree rings can also assess how climate change is affecting intrinsic water‐use efficiency (iWUE) and other physiological measures of tree performance (Silva and Anand 2013) through isotopic analyses. iWUE reflects the amount of water required for a tree to grow a set amount and is calculated as a ratio between assimilation and stomatal conductance. The abundance of δ^13^C and therefore iWUE in plant tissues is mainly driven by water, temperature, light, nutrient availability, and *C*_a_ (van der Sleen et al. 2017). Recent studies, have shown that iWUE has increased worldwide (Andreu‐Hayles et al. 2011; van der Sleen et al. 2015; Weiwei et al. 2018). Specifically, in a metanalysis of trees growing in diverse ecosystems across the United States, researchers found that iWUE has increased by 35% in the last 30 years (Guerrieri et al. 2019).

In this study, we used dendrochronology methods to determine the age structure and analyze annual tree growth increments and iWUE in three of south Florida's dominant tree species: *Taxodium ascendens* , *T. distichum*, and *Pinus elliottii*. Specifically, we used measurements of annual ring widths to assess how climate and hydrology are affecting tree growth rates in Big Cypress National Preserve (BICY) over extended temporal and landscape‐scales. In addition, we used stable isotopes to estimate iWUE and test how iWUE differs between species and is changing through time. We then tested the effects of climatic variables and hydrology on the iWUE of each tree species.

This project aimed to test four a priori research hypotheses: H1—Growth rates of *T. ascendens* , *T. distichum, and P. elliottii* are negatively related to temperature and VPD. In contrast, tree growth is positively related to precipitation and standing water levels because more water can extend the growing season. H2—Given their affinity for wet environments, growth rates are higher in *Taxodium* spp. trees that are closer to the areas of deeper water such as cypress domes and strands. In contrast, growth rates in *P. elliottii* will be higher in trees that are farther from inundated areas because of their affinity for drier conditions. H3—Since our study sites are near the warmest, southernmost limit of the three species' ranges, growth rates are decreasing through time in all three tree species as temperatures increase. And lastly, H4—The iWUE of the three tree species is increasing over time as the trees respond to increased evaporation and greater water loss caused by climate change coupled with the effects of rising *C*_a_ and “carbon fertilization.” By testing these hypotheses and characterizing the growth rates and water‐use efficiencies of *T. ascendens, T. distichum*, and *P. elliottii* , along with their relationships with climate and hydrologic variables, we hope to provide information that can be used to help guide ongoing and future land management directives, and climate change mitigation and adaptation measures in these important species and more broadly across south Florida's natural ecosystems.

## 2 Methods

### 2.1 Study Area

**FIGURE 1:**
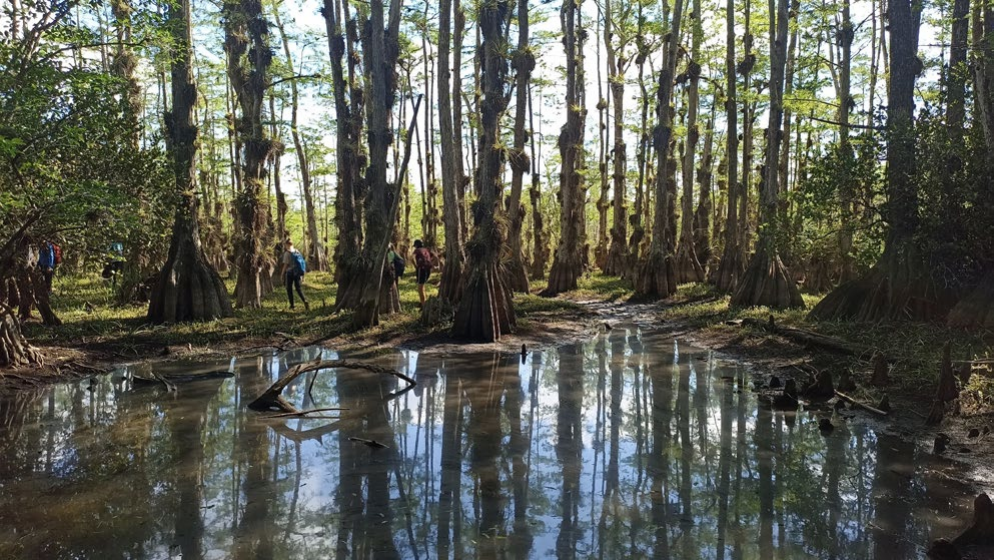
Interior view of a cypress dome dominated by *Taxodium distichum* during the dry season at Big Cypress National Preserve. Photo by Manuel Bernal‐Escobar.

This study was conducted in south Florida's Big Cypress National Preserve (BICY), which encompasses part of the western Everglades ecosystem and experiences seasonal inundation followed by annual dry periods (Hamann and Aitken 2013). There are five main plant communities in this ecosystem including hardwood hammocks, cypress forests, prairies, pinelands, and mangrove systems (Muss et al. 2003). *Taxodium distichum* establishes around deep depressions creating cypress “domes” (Figure 1), *T. ascendens* grows in ecotones between cypress domes and pinelands, also known as cypress prairies, and *P. elliottii* dominates the drier, upland pinelands. Geologically, our study area in BICY is on top of limestone with shallow, unconsolidated, nutrient‐poor soils which encompass rocks, carbonate marls, sands, organic soils, and peats (Duever et al. 1979). Generally, *T. ascendens* grows in shallow sandy marls, *T. distichum* grows in depressions filled with peat or marls, and *P. elliottii* grows in sandy soils (Duever et al. 1979). BICY has a gentle slope from northeast to southwest; its maximum elevation is 7 m asl and is fed mainly by annual precipitation during the wet season, with additional inflow from Lake Okeechobee on its eastern side (Duever et al. 1979). Its geological features and variable water levels create a patchwork of wet, cypress‐dominated depressions and slightly higher, seldom flooded pinelands.

**FIGURE 2:**
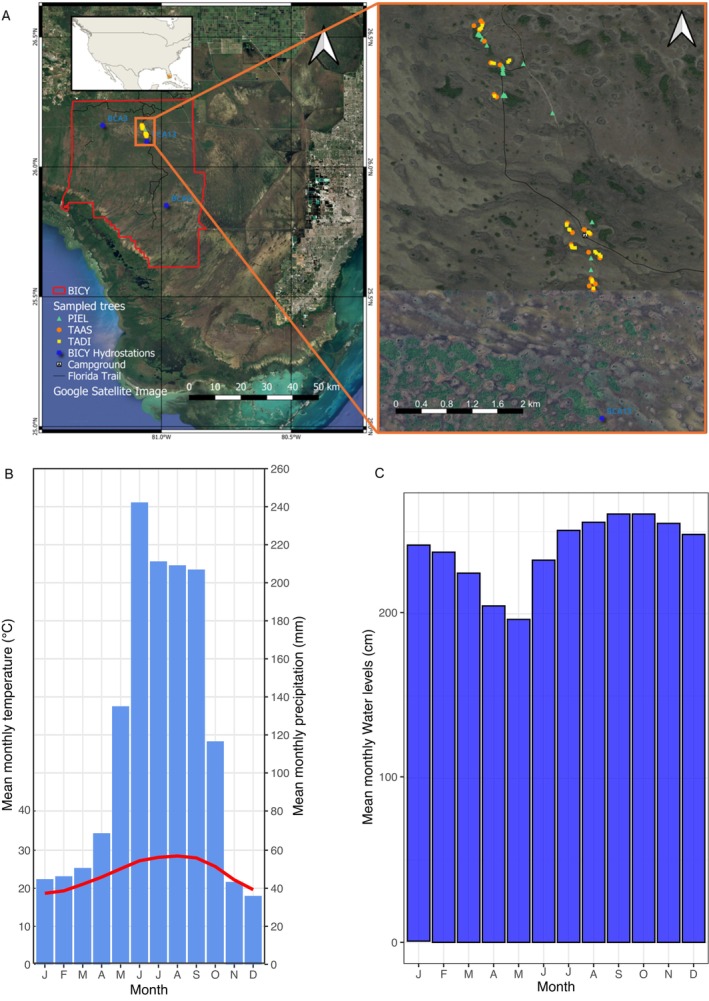
(A) Map of Big Cypress National Preserve (BICY) and collection locations. TAAS ( *T. ascendens* ), TADI ( *T. distichum* ), and PIEL ( *P. elliottii* ). (B) Climograph showing mean monthly temperature and precipitation for the collection site from 1895 to 2022 (PRISM Climate Group and Oregon State University 2023). The rainy season occurs from April to October, and the dry season occurs from November to March. (C) Shows the mean monthly water levels from station BCA 6 from 1990 to 2021 (EDEN project, gathered from sofia.usgs.gov).

The wood samples used in this study were collected from living trees in the Nobles Grade region in the Mullet slough unit of BICY (Figure 2A). In 2022, we sampled *Taxodium* spp. around 10 cypress domes and *P. elliottii* in nine pinelands along the Florida Trail and between 0.7 and 5 km south of the I‐75 highway. We included in our samples trees growing at different distances from the center of the domes for *Taxodium* spp. and pine trees at different distances to the edge of the pinelands so that we could assess the effect of the relative position within each dome or pineland on the growth rates of each species.

The mean annual temperature in BICY for the period 1895–2022 is 24°C, and the mean annual precipitation over that same period is approximately 1500 mm. Summers (April—October) are hot and wet with temperature and precipitation maximums (27°C and 260 mm/month) occurring during this period, while winters (November—March) are cooler and drier with temperature and precipitation reaching minimums (18°C and 40 mm/month) during this period (Figure 2B). During the last century, mean annual temperature, total annual precipitation, and VPD have increased at different rates, but changes have been more dramatic since the late 1970's (Figure S1) (PRISM Climate Group and Oregon State University 2023; Meteoblue 2025). In general, conditions have changed to be hotter and drier during the dry season, but to be wetter during the summers due to more frequent storms (Climate Change Response Program 2024). Seasonal water depths range from 1.3 to 2.8 m in the longest running water gauge (BCA 6) near our study area. Although gauges BCA 3 and 13 are closer to the sampling site we selected gauge BCA 6 because it had a longer recorded period (i.e., 1990 to 2021) gathered from EDEN project (Telis 2006). It is important to note that gauge BCA 6 is located ~32 km south of our location in a cypress strand that seldomly dries out (Figure 2C). Water levels at BCA 6 reach their monthly minimum value (< 2 m) during April and May, whereas the highest levels occur during September and October. Although precipitation is positively related to water depth, it only explains 5% of variation, suggesting that there are other factors affecting the quantity of water flowing through the system (Figure S2).

We collected wood samples (cores) of 22 pond cypress ( *T. ascendens* ), 26 bald cypress ( *T. distichum* ), and 26 slash pine ( *P. elliottii* ) trees. The sample trees were healthy individuals with diameter at sample height (DSH) > 4.5 cm. We sampled trees between 0.5 and 1.5 m above the ground, trying to get the oldest rings in the trees at the lowest possible height but avoiding buttresses and standing water (in some cases water levels reached ~1 m during sample collection). We collected two cores from each sample tree using a sharpened and sterilized wood increment borer (Haglof 5.15‐mm‐diameter) following standard techniques (Speer 2010). Samples were taken to the University of Miami (Coral Gables, Florida), where they were dried and prepared following standard protocols (Speer 2010). Each sample was then scanned with a high‐resolution flatbed scanner at 2400 dpi (Epson V800 Photo).

### 2.2 Development of Tree‐Ring Chronologies and Quality Quantification

We manually identified and marked the boundaries of annual growth rings on all cores in scanned images using the CooRecorder program (Cybis Elektronik & Data AB; http://www.cybis.se/forfun/dendro/). An interactive plotting tool was employed to estimate the age and width of growth rings from the bark to the pith with a maximum precision of 0.001 mm (Maxwell and Larsson 2021; Stahle 1999). Dating results were validated using the program COFECHA v 6.06 (Holmes 1983) and we standardized our samples with the program ARSTAN v 49v1bWin (Cook 1985). Standardization for each species involved applying an age‐dependent spline with an initial 20‐year kernel to eliminate ontogenetic effects from the analyzed growth rates (Melvin et al. 2008; Peters et al. 2015). After that, we calculated a ring width index (RWI) as the ratio between each species raw data and its fitted model, and finally, we calculated the mean RWI residual chronology for each species with an autoregressive model (Brockwell and Davis 2016) and a bi‐weighted mean (Mosteller and Tukey 1977). We then computed the mean sensitivity (MS), series intercorrelation (r_xy_), and the expressed population signal (EPS) (Bunn et al. 2022; R Core Team 2022). MS is a measure of year‐to‐year variation in tree‐ring series on a scale of 0 to 2 (Bunn et al. 2013) such that values close to zero and indicate low variability, values > 0.2 and < 0.6 indicate ideal sensitivity for climate reconstruction, and values > 0.6 suggest excessive sensitivity preventing reliable crossdating (Biondi and Qeadan 2008b). *r*_xy_ represents the average of all correlations between individual series (*x*) and the species mean chronology (*y*). EPS is a metric that indicates how much of the signal in the chronology is shared within the sampled population (Speer 2010; Wigley et al. 1984).

### 2.3 Relationships Between Tree‐Ring Growth and Climate Variables

We downloaded climate data at a 4 km grid cell resolution from the PRISM database (PRISM Climate Group and Oregon State University 2023) using the centroid of our sampling site (26.1192° N, −79.0551° W). Data included total monthly precipitation, minimum monthly temperature, mean monthly temperature, maximum monthly temperature, minimum monthly vapor pressure deficit (VPD), and maximum monthly VPD from January 1895 to January 2022 (Figure S1). To estimate the effect of hydrology on tree growth and ecosystem flood dynamics, we used monthly water depth data collected from station BCA 6 developed by the EDEN project from 1990 to 2021 gathered from sofia.usgs.gov (Figure 2C, Figure S1B) (Telis 2006).

To assess how standardized tree growth rates were influenced by climate, we computed Pearson correlations between each species' RWI mean chronology (i.e., standardized growth rates) and the climate and water depth of the current and preceding year. Specifically, we used monthly mean, maximum, and minimum temperatures, total monthly precipitation, monthly maximum and minimum VPD, and monthly water depth. We calculated averages for temperatures and VPD and sums of precipitation for winter and summer seasons (i.e., January–March and June–September). Correlations with the previous year's conditions were included since those conditions can influence growth in the subsequent year (Fritts 1976). We conducted climate–growth correlation analyses using the *treeclim* package (Zang and Biondi 2015), which estimates univariate Pearson product–moment correlation coefficients between tree‐ring indices and climate variables (Biondi and Waikul 2004). Statistical significance was evaluated using stationary bootstrap confidence intervals as implemented in *treeclim*.

### 2.4 Changes in Tree Growth Rates Through Time

To assess how tree growth rates of each species changed over time, we converted the ring widths to basal area increments (BAI) with the following equation in the dplR library in R (Bunn et al. 2022):

(1) $$\mathrm{BAI}={\pi \;r}_{t}^{2}-{\pi \;r}_{t-1}^{2}$$

where *r*_*t*_ is the radius at the end of the annual increment, a *r*_*t‐1*_ is the stem radius at the beginning of the annual increment (Biondi and Qeadan 2008a) Then, to account for effects of ontogeny we performed an Age Class Isolation analysis, such that the BAI from same‐aged trees are compared over time (Rozendaal et al. 2010; Peters et al. 2015). Specifically, we calculated the average BAI of all individual trees within 5‐year age classes centered at 10‐year age increments (i.e., 7.5–12.5, 17.5–22.5, 27.5–30.5, …, 117.5–122.5 years old) and looked at relationships between the growth of trees in the age class versus the calendar years at which the trees achieved that age (Peters et al. 2015).

### 2.5 Tree Growth Rates and Spatial Relationships

To explore how tree growth rates varied across the landscape, we conducted linear regression analyses to examine relationships between BAI and various spatial variables. To account for ontogenetic effects, we applied an age‐dependent spline with an initial 20‐year kernel to the BAI data and used the resulting residuals as our response variable in subsequent models. Using remotely sensed imagery and QGIS (version 3.40.0), we measured the distance from each sampled tree to the nearest point of Interstate 75 (“Alligator Alley”), a highway with multiple construction phases between 1920 and 1992. This road is known to disrupt and alter water flow south of its location (Sklar et al. 1999; Fling et al. 2024). For *Taxodium* spp., we also calculated the distance to the center of the nearest cypress dome, while for *P. elliottii* , we measured the distance to the nearest edge of the surrounding pineland. We then fit linear models to assess: (1) the relationship between standardized growth rates for all species and distance to the road, (2) the relationship between standardized growth rates of *Taxodium* spp. and distance to the center of the nearest dome, and (3) the relationship between standardized growth rates of *P. elliottii* and distance to the pineland edge.

### 2.6 Isotopic Analysis

Once all ring widths were measured and crossdated, we used a surgical scalpel and a stereomicroscope to extract subsamples of wood at 10‐year increments (i.e., 2020, 2010, 2000, 1990, 1980, 1970, …, 1890, 1880) and after that every 20 years (i.e., 1860, 1840, 1820, 1800). Centered on each ring, we collected ~0.5 mg of wood. We collected samples from 16 individuals of each species. As each tree has a different age, the quantity of samples per individual changes. In total we collected 167 samples of *T. ascendens* , 133 samples of *T. distichum* , and 116 samples of *P. elliottii* , for a total of 420 samples. We followed van der Sleen et al. (2017) for sample preparation.

We submitted wood samples to the University of Arizona's Environmental Isotope Lab where samples were analyzed for δ^13^C as bulk wood in a continuous‐flow gas‐ratio mass spectrometer (Finnigan Delta PlusXL) coupled to an elemental analyzer (Costech). Standardization was based on acetanilide for elemental concentration, NBS‐22 and USGS‐24 for δ^13^C, precision was better than ±0.10 for δ^13^C (Wang et al. 2020; Yanay et al. 2022). The resultant isotopic ratios were used to estimate iWUE of the trees at the time of wood formation using isocalcR (Mathias and Hudiburg 2022). We calculated iWUE using the “simple” formulation within the package, specifying that the samples came from wood tissue so the parameters in the equation reflect the post‐photosynthetic fractionations that have taken place (i.e., 2‰) (Belmecheri and Lavergne 2020; Mathias and Hudiburg 2022; Siegwolf et al. 2022). Specifically, the carbon isotope composition (δ^13^C, in ‰) of the samples was calculated as follows:

(2) $$\delta^{13}C_{\text{Sample}}=\left(\frac{R_{\text{Sample}}}{R_{\text{Standard}}}-1\right)*1000$$

where *R*_sample_ is the ^13^C/^12^C ratio of the sample and *R*_standard_ is the ^13^C/^12^C ratio of an established standard. Discrimination against the heavier ^13^C (Δ^13^C) is then calculated as:

(3) Δ13C=δ13Ca−δ13CSample1+δ13CSample1000

where δ^13^C_a_ is the δ^13^C of atmospheric CO_2_ in the northern‐hemisphere tropics at the year of wood formation (Graven et al. 2017). CO_2_ concentration within the leaf intercellular spaces (*C*_i_) is then estimated through the relationship:

(4) Δ13C≈a+b−aCiCa

where *a* (≈4.4‰) represents to the isotopic discrimination of atmospheric CO_2_ (*C*_a_) entering the leaf intercellular spaces due to the slower diffusion of ^13^CO_2_ compared to ^12^CO_2_ through stomata, and *b* (≈27‰) represents the isotopic discrimination of CO_2_ during carboxylation by Rubisco (Farquhar et al. 1982). Historic *C*_a_ estimates for subtropical regions were taken from Belmecheri and Lavergne (2020) and Mathias and Hudiburg (2022).

The relative iWUE at the time of the sampled wood formation was then estimated as:

(5) iWUE=Ags=Ca−Ci1.6

where *A* is the rate of CO_2_ assimilation and *g*_s_ is the rate of leaf stomatal conductance.

Then, to control for ontogeny effects, we fit a linear model between iWUE and age (Brienen et al. 2017; van der Sleen et al. 2017) and extracted the residuals to evaluate changes in iWUE over time and relationships with climatic variables. All analyses were done in R v.4.2.2 (R Core Team 2022).

## 3 Results

**FIGURE 3:**
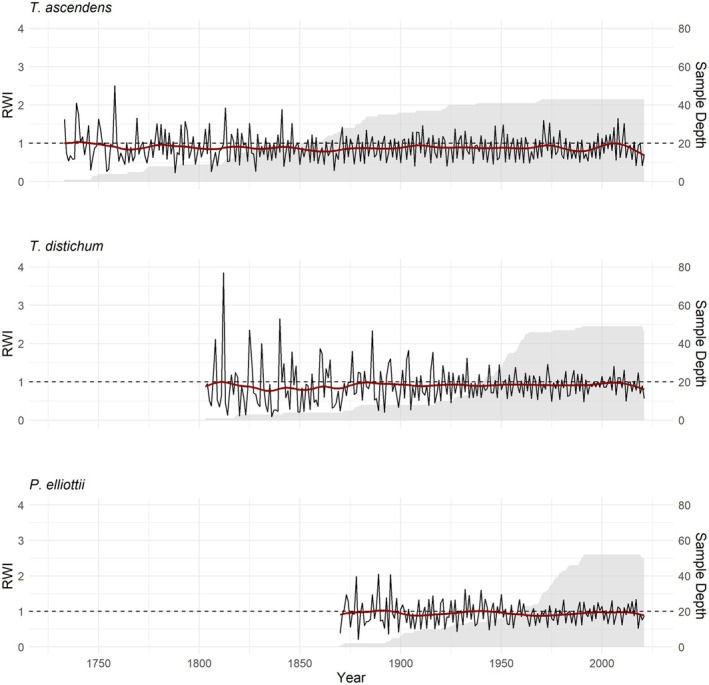
Ring width index (RWI) constructed from the residual chronology and sample depth from Big Cypress National Preserve. Left *Y* axis shows RWI in black and the adjusted spline function in red. Right *Y* axis shows sample depth on a gray background.

Ages for the three focal tree species ranged from 52 to 289, 46 to 219, and 46 to 162 years, for *T. ascendens* , *T. distichum* , and *P. elliottii* , respectively. The mean age of the sampled individuals was 174 years old for *T. ascendens* , 89 years old for *T. distichum* , and 69 years old for *P. elliottii*. In constructing our growth chronologies, we found series intercorrelations > 0.40 for the three species, and mean sensitivities of 0.717 for *T. ascendens* , 0.672 for *T. distichum* , and 0.569 for *P. elliottii* (Figure 3, Table S1). On average, *T. ascendens* exhibited a mean radial growth of 0.83 mm/year and displayed its strongest growth–water level correlations in June; *T. distichum* grew on average 2.65 mm/year showing significant growth–water level correlations from May to October; and *P. elliottii* averaged 3.06 mm/year, with significant growth–water level correlations during April and May.

We did not find significant relationships between tree growth rates and the current year's climatic variables. We found that tree growth rates were weakly related to the previous year's climate variables such as total monthly precipitation; monthly mean, minimum, and maximum temperature; and monthly minimum and maximum VPD. Growth rates of *T. ascendens* showed a significant positive correlation with precipitation of the previous year's March (*r* = 0.20). There were no significant correlations between growth rates of *T. distichum* and precipitation. *P. elliottii* showed significant positive correlations with precipitation of January (*r* = 0.21) and April (*r* = 0.25) of the previous year, and a significant negative correlation with previous June precipitation (*r* = −0.20) (Figure S3). We found significant positive correlations between *T. distichum* growth and previous August maximum temperature (*r* = 0.22) and marginally significant correlation between *P. elliottii* growth and the previous April minimum temperature (*r* = 0.16) (Figure S4). We also found a significant positive correlation between *T. distichum* and previous year's August maximum VPD (*r* = 0.18) and a negative correlation between *P. elliottii* and the previous April maximum VPD (*r* = −0.13) (Figure S5).

**FIGURE 4:**
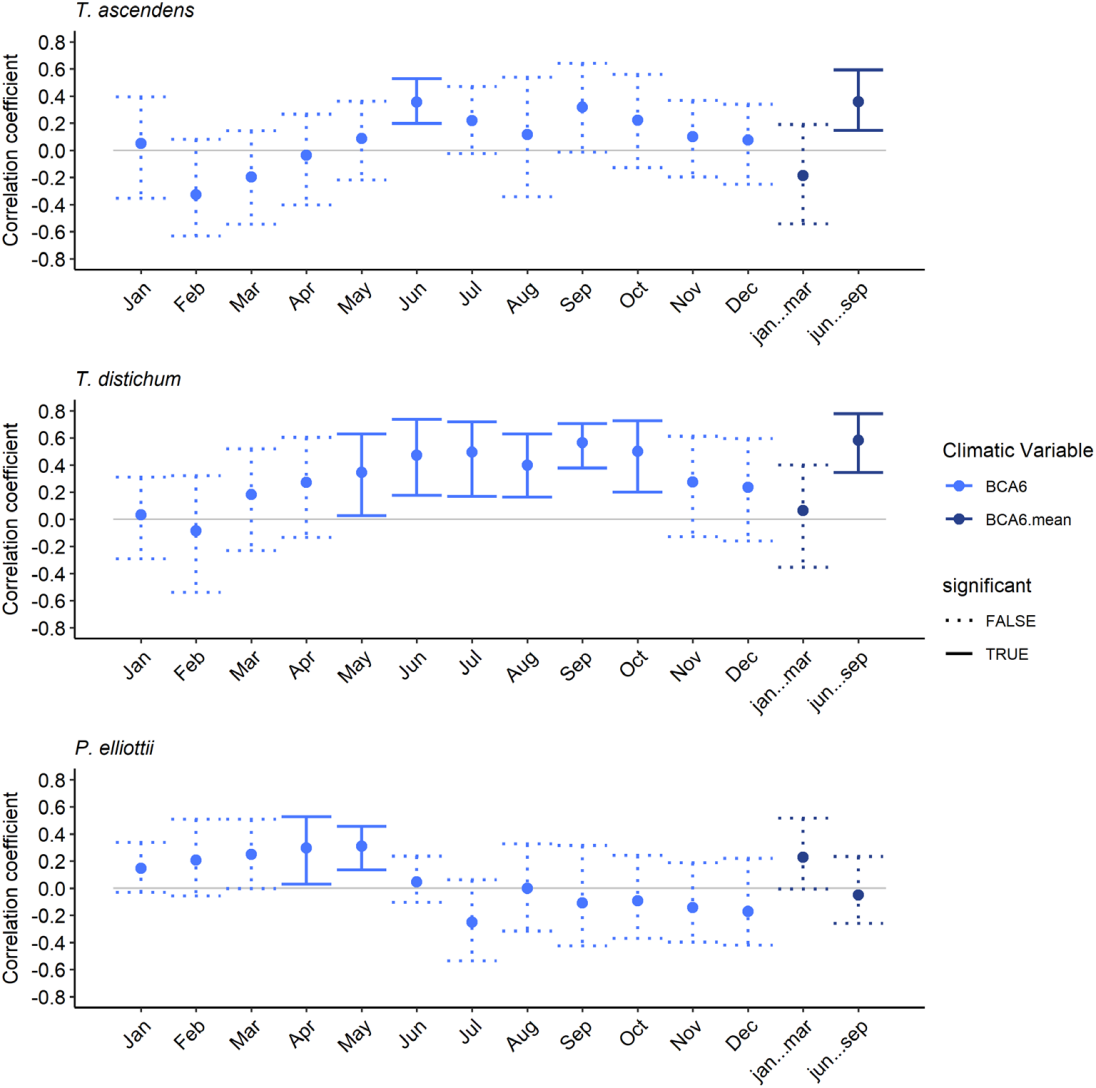
Pearson correlation values between *T. ascendens* , *T. distichum* , and *P. elliottii* RWI chronologies and previous year water level monthly data from station BCA 6 from 1990 to 2021.

Tree growth rates had stronger relationships with water levels from April to October. Specifically, *T. ascendens* growth was correlated positively with June (*r* = 0.34) and mean June–September water levels (*r* = 0.35), growth of *T. distichum* was correlated positively with water levels from May (*r* = 0.31) until October (*r* = 0.49) and the mean from June–September (*r* = 0.57), and growth of *P. elliottii* was correlated positively with April (*r* = 0.29) and May (*r* = 0.30) water levels (Figure 4). We analyzed the response to water depth over time, and we found that the relationships are stable between 1990 and 2021 (Figure S6). However, since the water level records start in 1990, we could not evaluate whether the road construction had a significant effect on altering water flow and therefore tree growth.

**FIGURE 5:**
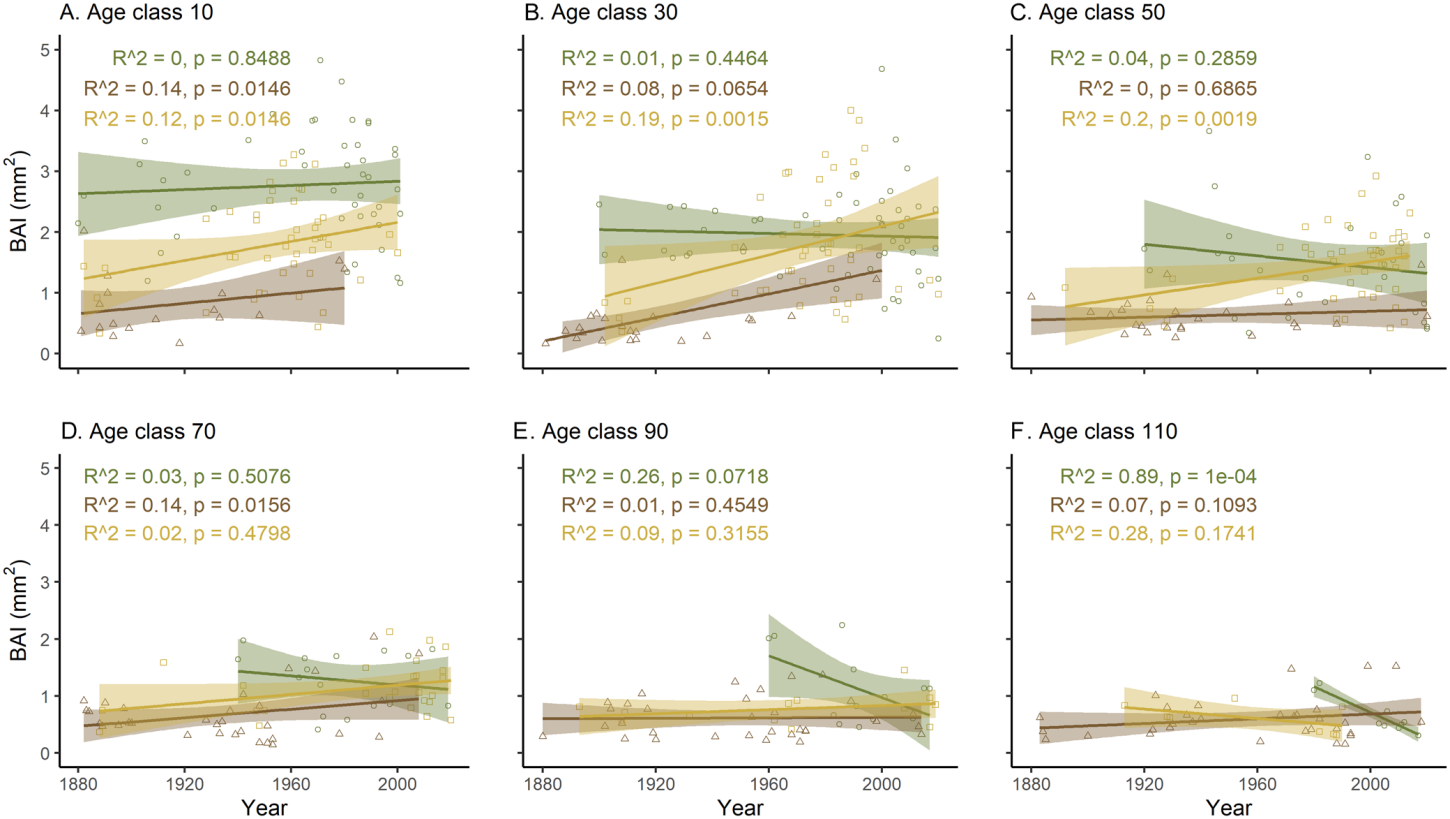
Age class analysis for: (A) Age class 10, (B) Age class 30, (C) Age class 50, (D) Age class 70, (E) Age class 90, (F) Age class 110. *Y* axis: Basal Area Index (mm^2^), *X* axis: time in years. *Taxodium ascendens* in brown, *T. distichum* in yellow, and *P. elliottii* in green.

Based on our Age Class Isolation analysis, we found that tree growth rates are changing through time. Both *Taxodium* species showed increasing growth rates through time in younger aged individuals. *T. distichum* increased its growth rates through time up to age class 50 (*R* ^2^ = 0.2 *p* = 0.0019), whereas *T. ascendens* increased its growth rates up to age class 40 (*R* ^2^ = 0.08, *p* = 0.0682) (Figure 5, Figure S7). Another significant and positive period of growth for *T. ascendens* was age class 70 (*R* ^2^ = 0.14, *p* = 0.0156). *P. elliottii* revealed no changes in growth rates through time in young individuals but decreasing growth rates over time in individuals in age clases 40 (*R* ^2^ = 0.09, *p* = 0.0479), 60 (*R* ^2^ = 0.16, *p* = 0.0564), 90 (*R* ^2^ = 0.26, *p* = 0.0718), and this trend is held until age class 120 (*R* ^2^ = 0.59, *p* = 0.0448) (Figure 5, Figure S7).

**FIGURE 6:**
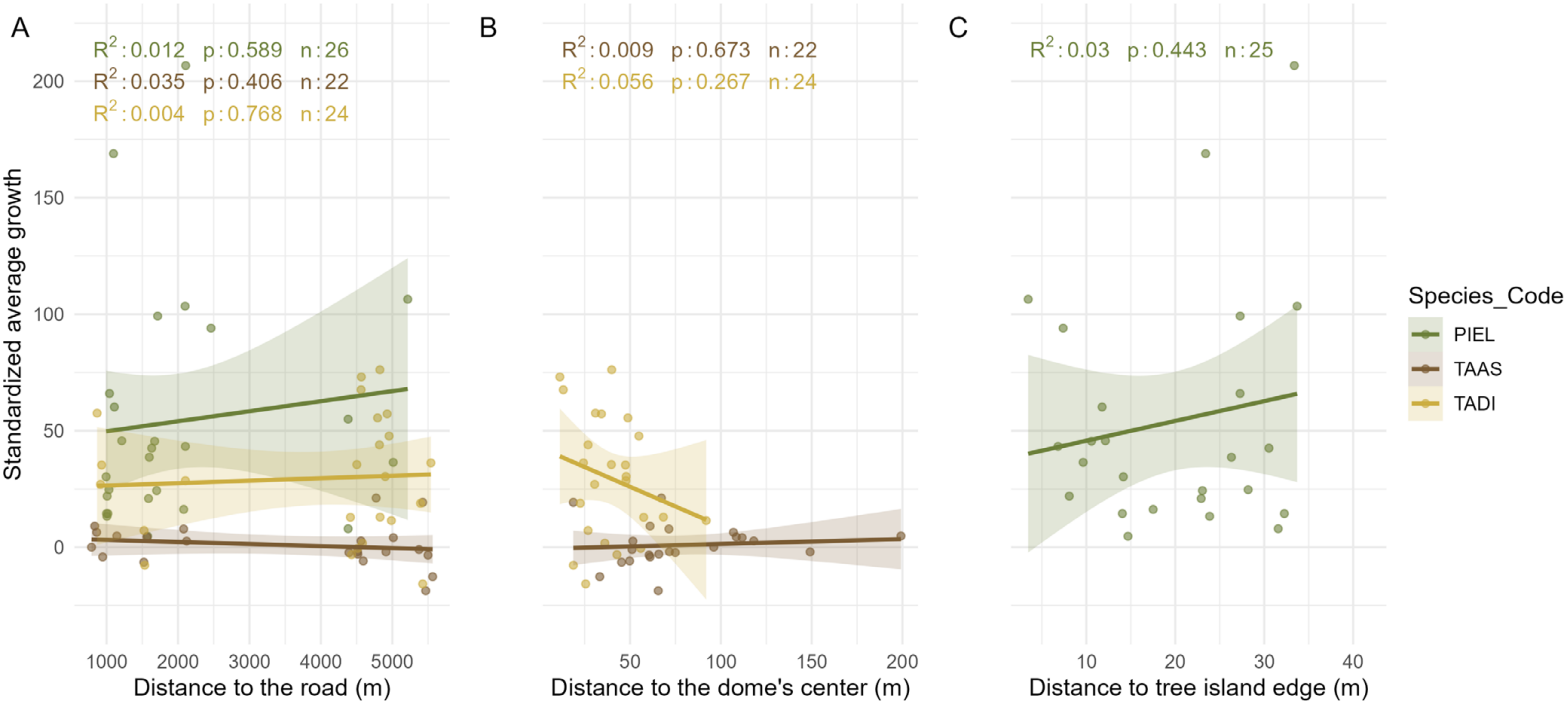
(A) Regression between mean tree growth rates and distance to the road (Interstate‐75). (B) Regression between mean growth and distance to dome ( *T. ascendens* and *T. distichum* ). (C) Regression between mean growth and distance to the edge of the pinelands ( *P. elliottii* ).

We found that the relative location of the trees within the landscape did not affect their growth rates. Specifically, *T. distichum* and *T. ascendens* growth rates did not change when they were farther from the center of a cypress dome (*R* ^2^ = 0.05, *p* = 0.267 and *R* ^2^ = 0.00, *p* = 0.673), respectively. Similarly, *P. elliottii* growth rates did not change when they established farther from the pineland edges (*R* ^2^ = 0.03, *p* < 0.443). We did not find any significant trend between the species mean tree growth rates and the distance to the I‐75 highway (Figure 6).

**FIGURE 7:**
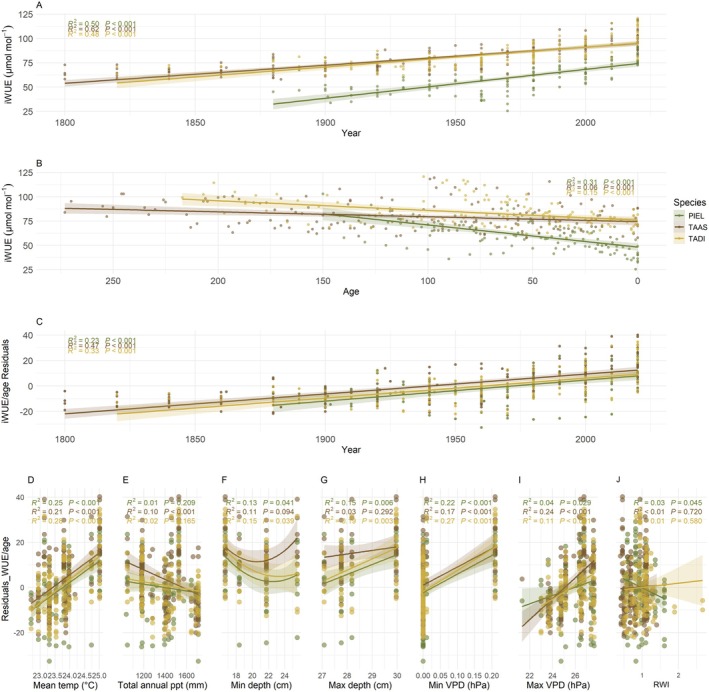
(A) Shows changes in iWUE over time broken down by species and lower panels show iWUE relationship with climatic variables, (B) Changes in iWUE versus tree age, (C) iWUE versus age residuals over time, (D) iWUE age residuals versus mean temperature (°C), (E) versus total annual precipitation (mm), (F) versus minimum depth (cm), (G) versus maximum depth (cm), (H) versus maximum VPD (hPa), (I) versus maximum VPD (hPa), (J) versus RWI (Ring Width Index).

We found that iWUE was relatively stable in all three species from 1800 until around 1930, then increased rapidly over time (Figure 7A). There was a significant effect of tree age on iWUE, with older individuals having lower efficiency in all three species (Figure 7B). After accounting for this age effect, the three species all exhibited similar average iWUE versus year relationships with iWUE increasing steadily and significantly through time (Figure 7C). In all three species, this increase was associated with increasing mean annual temperatures (*p* < 0.001) (Figure 7D) and increasing carbon dioxide concentrations *C*_a_ (*p* < 0.001) (Figure S8B). We found that total annual precipitation was negatively related to iWUE in *T. ascendens* (*p* < 0.001), but not in *T. distichum* or *P. elliottii* (Figure 7E). We found that minimum water depth had a negative relationship with iWUE, and maximum depth had a positive relationship with iWUE in *T. distichum* (*p* < 0.039 and *p* < 0.003, respectively) and *P. elliottii* (*p* < 0.041 and *p* < 0.006, respectively), but no relationship with iWUE in *T. ascendens* (*p* < 0.094 and *p* < 0.292, respectively) (Figure 7F,G). Moreover, we found that minimum and maximum VPD had significant positive effects on iWUE in all species; that is, *T. ascendens* (*p* < 0.001 and *p* < 0.001, respectively), *T. distichum* (*p* < 0.001 and *p* < 0.001, respectively), and *P. elliottii* (*p* < 0.001 and *p* < 0.029, respectively) (Figure 7H,I). Lastly, we found that iWUE of *T. ascendens* and *T. distichum* species were not correlated with growth rates (*p* < 0.720 and *p* < 0.580, respectively), whereas in *P. elliottii* , iWUE was negatively related with growth (*p* < 0.045) (Figure 7J).

## 4 Discussion

Using dendrochronology and stable isotope analyses, we examined the changes in annual growth rates and iWUE over temporal and landscape scales within three of south Florida's dominant tree species ( *Taxodium ascendens* , *T. distichum* , and *P. elliottii* ), all growing in the southern/hotter ends of their distributions. We observed that changes in tree growth rates over time were strongly influenced by water depth. Furthermore, we found strong increases in iWUE over time after controlling for age, associated with rising air temperatures, increasing atmospheric carbon dioxide concentrations, changes in precipitation, and VPD.

### 4.1 Tree Growth Rates in Relation to Water Levels and Climatic Variables

The annual growth rates of *T. ascendens* had a significant positive correlation with June and summer mean water levels (June—September). The growth of *T. distichum* had an even stronger positive relationship with water levels, with the positive effects extending until October. These positive relationships between standing water and tree growth rates highlight the preference of cypress trees for wetter conditions (Figure 4, Figure S6). The relationships also suggest that under optimal hydrologic and climatic conditions, trees grew more rapidly than during other time periods when those conditions did not exist. The growth in *Taxodium* spp. peaks along with the timing of standing water, as higher water levels lead to more surface flooding and may benefit most of the growing season into early fall. More water in the system may also mean cooler water with more dissolved gases, including oxygen, which may increase aeration in the roots and lead to faster growth (Sullivan et al. 2014). Unexpectedly, August mean and maximum temperatures, together with August maximum vapor pressure deficit (VPD), were positively associated with radial growth in *T. distichum* . Although elevated temperatures and VPD are often expected to constrain growth via stomatal closure, this response may reflect the species' high tolerance to warm conditions when water availability is not limited. Under flooded or high–water table conditions, increased temperatures may enhance photosynthetic rates and cambial activity, allowing growth to be maintained or even accelerated despite higher atmospheric demand.

There were significant positive correlations between *P. elliottii* growth and water levels in the previous year's April and May (Figure 4, Figure S6). These two months are the end of the dry season; therefore, increases in water level at this time may shorten the dry season and promote earlier onset of growth in pinelands. However, contrary to our initial predictions, growth rates of *P. elliottii* correlated positively with minimum temperature of the previous year's April, indicating that warmer early season temperatures potentially allow for earlier growth and a longer growing season as well. On the other hand, growth rates of *P. elliottii* , which grows in the drier pinelands, were negatively correlated with the previous year's April maximum VPD, in line with our initial hypotheses and findings that greater April precipitation increases *P. elliottii* growth.

The growth of *T. distichum*, which grows primarily in or around cypress domes with deeper water, correlates positively with previous August maximum VPD (Keeland and Sharitz 1995; Zheng et al. 2023). These trees are not typically water limited, so the higher VPD could allow for greater transpiration, which in turn could help to cool leaves and allow for faster rates of photosynthesis (Grossiord et al. 2020). Conversely, other studies have shown that rising VPD due to climate change can negatively affect tree growth (Bauman et al. 2022; Sanginés de Cárcer et al. 2018; Yuan et al. 2019). Furthermore, in contrast to *T. distichum* , the growth of *T. ascendens* , which inhabit the transition zones between pinelands and cypress domes, was not related to maximum VPD, suggesting that it is adapted to thrive in harsh habitats by regulating its growth rates.

The relatively weak relationships between previous and current year climate and tree growth rates of our focal trees might be due to a combination of several factors. Sah et al. (2014) found that water levels in flooded ecosystems can drive changes in vegetation composition and fitness. Thus, the natural water retention of this ecosystem could potentially override other kinds of environmental cues. Second, trees growing at their southern range extents may be more sensitive to species interactions (Paquette and Hargreaves 2021) and less sensitive to climate than more northern populations, which may indicate non‐linear responses to warming at the species' range margins (Zampieri et al. 2024). Third, trees may be acclimating to changes in climate through adjustments in the morphology or physiology of leaves or other tissues such as iWUE, as discussed below (Silva and Anand 2013). Fourth, other disturbances such as fires (Harley et al. 2013; Duever et al. 1979), hurricanes (Ortegren and Maxwell 2014), selective logging (Reategui‐Betancourt et al. 2025), or the construction of roads and canals that have been occurring since the 1900s may have altered the natural water flow (Duever and McCollom 1987; Duever et al. 1979) and affected tree health and ecophysiological performance producing novel trends seen herein.

### 4.2 Changes in Intrinsic Water Use Efficiency (iWUE) Over Time Are Driven by Mean Temperature, VPD, and *C*_a_

All three tree species showed a steady increase in intrinsic water‐use efficiency (iWUE) over time, confirming our hypothesis that rising temperature, greater water loss, and increasing CO_2_ concentrations enhance iWUE. This trend aligns with observations from diverse ecosystems worldwide (Andreu‐Hayles et al. 2011; Weiwei et al. 2018; van der Sleen et al. 2015). Also in accord with our hypothesis and other studies (Frank et al. 2015; Grossiord et al. 2020; Lu et al. 2024; Wang et al. 2018), the iWUE of our focal trees was positively related to increases in mean temperature, minimum and maximum VPD, and atmospheric carbon concentration (*C*_a_). This suggests that hotter temperatures, drier air, and higher CO_2_ concentrations are forcing our focal species to be more efficient at using water. In contrast, the relationships between iWUE and water‐related climatic variables (i.e., total annual precipitation and minimum and maximum water depth) differed between species. In *T. ascendens* , iWUE was negatively related to precipitation but was not related to water level depths, whereas iWUE of *T. distichum* and *P. elliottii* had no relation with total annual precipitation but significant positive relationships with minimum and maximum water depths. The positive relationships between maximum water depths and iWUE in *T. distichum* and *P. elliottii* may suggest that years with above‐average water levels in the ecosystem can induce stress in these trees, prompting an increase in their iWUE, following Shelford law's which suggest that stress can be induced by excess or absence of a resource (Shelford 1911). Conversely, the non‐linear relationship between minimum water depth and iWUE in *T. distichum* and *P. elliottii* indicates that these species experience stress under both excessively low and high‐water conditions, leading to increased iWUE. This pattern aligns with findings from Li et al. (2022), who reported that soil water contributions exhibited significant quadratic correlations with water table depth under shallow water table conditions.

The iWUE of the *Taxodium* species was not related to growth rates in our study. These patterns may stem from *Taxodium* species' adaptations to seasonally dry‐flooded ecosystems, such as annual defoliation during the dry season (USDA 2025) and specialized roots that facilitate respiration during flooding (Thurman and Crisman 2023). In contrast, *P. elliottii* exhibits a decrease in iWUE during years of high growth, suggesting an increased risk of hydraulic stress when carbon gain is achieved at the expense of water‐use efficiency (Siegwolf et al. 2022). Similar to *P. elliottii* , Gea‐Izquierdo et al. (2021) found that *Pinus sylvestris* and *Quercus pyrenaica* exhibited declining growth rates despite increasing iWUE, suggesting increasing constraints on leaf gas exchange. Likewise, Wang et al. (2024) reported significant iWUE increases for *Juniperus tibetica* (22%) and *Picea balfouriana* (26%) on the Tibetan Plateau, in agreement with the increasing iWUE trends observed over time in all three of our focal species. However, while *J. tibetica* showed concurrent increases in growth and iWUE, growth responses in *P. balfouriana* diverged from this pattern, with declining growth despite rising iWUE, closely paralleling the response observed in *P. elliottii* in our study. In *P. balfouriana* , iWUE increases were primarily driven by reductions in stomatal conductance rather than enhanced photosynthesis, reinforcing our interpretation that moisture limitation, and not carbon supply can constrain growth even as iWUE increases.

Wetland trees often respond differently than those on dry land. Previous studies in south Florida's Everglades showed that increased wet‐season precipitation can trigger native plants to either reduce stomatal conductance or boost photosynthetic assimilation (Ewe and Sternberg 2003; Sauter 2013). When flooding raises water above the soil surface, creating anoxic root conditions, both water and nutrient uptake are disrupted (Anderson et al. 2005). Therefore, anthropogenic alterations in water flow, such as those produced by the construction of roads and canals built since the early 1900s, may have exacerbated root hypoxia, leading to reduced hydraulic conductivity (Else et al. 2001), elevated abscisic acid levels (Wei et al. 2025; Wurms et al. 2023), and accumulation of metabolic toxins (Pedersen et al. 2017, Manghwar et al. 2024). These disruptions in root function may lead to a decline in stomatal conductance (Ewe and Sternberg 2003; Sauter 2013), ultimately reducing transpiration and sap flow. If this hypothesis holds true, the positive correlations between the iWUE of *T. distichum and P. elliottii* with minimum and maximum depths could indicate water stress resulting from rising water levels during wetter wet seasons.

These results indicate that *T. ascendens* is better adapted to drier, more stressful environments. Its inherently low growth rates likely confer an advantage under harsher conditions compared to the other two species. At the same time, these results highlight that *T. distichum* and *P. elliottii* are both sensitive to the amount of water at the soil surface. While *T. distichum* requires year‐round flooding to grow well, *P. elliottii* decreases growth if flooded for too long (Belmecheri et al. 2021). Together these results show that hotter, drier air, and soils have caused an accelerated increase in iWUE but differential responses on growth rates in the three species as a likely consequence of increasing *C*_a_ and climate change (Saurer et al. 2014).

### 4.3 Relative Location of Trees Within Domes and Pinelands do not Influence Growth Rates

In *T. distichum* , growth rates were not significantly influenced in trees located closer to the centers of cypress domes, contrary to our hypothesis that growth would increase with proximity to deeper water areas. This pattern likely reflects the limited hydrological gradient within domes, where trees, even those near the margins, generally have year‐round access to water. Because the domes rarely dry out, water availability is unlikely to constrain growth, and trees across the dome can maintain favorable moisture conditions and relatively cooler leaf and air temperatures for much of the growing season (Duever and McCollom 1987).

*T. ascendens* growth rates did not follow our hypothesis and were not influenced by the distance to the center of the dome. This highlights *T. ascendens* adaptations, specifically its slower growth rates, that allow it to cope with harsh substrates and mitigate additional stress like droughts and floodings (Zhang et al. 2020). These findings align with Duever and McCollom (1987), who reported that the oldest *T. ascendens* individuals in cypress swamps occur on marl soils or shallow soils over bedrock. Also, in accord with Duever and McCollom (1987), we observed extremely narrow annual rings in this species, frequently consisting of only one or two cell rows in the earlywood and latewood and yielding annual DBH increments of ~1 mm. This persistent slow growth may represent a stress‐tolerant strategy, whereby reduced growth rates lower physiological demands under limiting conditions, potentially facilitating long‐term survival and longevity in relatively small individuals (Duever and McCollom 1987).

Growth rates of *P. elliottii* were not higher in individuals located farther from pineland edges, contrary to expectations based on previous studies (Foster and Brooks 2001; Harley et al. 2015). Although this species does not thrive in water‐saturated soils (Harley et al. 2015), pinelands exhibit a pronounced nutrient availability gradient from their centers toward the edges (Espinar et al. 2011; Sullivan et al. 2014). In particular, nitrogen is depleted upstream of pinelands, and the N:P ratio increases significantly from upstream to downstream positions (Espinar et al. 2011). As a result, nutrients tend to accumulate near pineland edges, potentially promoting higher growth rates in these locations (Sullivan et al. 2014). These patterns suggest that growth of *P. elliottii* in pinelands may be more strongly limited by nutrient availability than by soil water saturation.

### 4.4 Growth Rates Through Time

Age Class Isolation analyses revealed that older *P. elliottii* trees have experienced declining growth over time, supporting our hypothesis that trees at the hotter end of their distribution will slow their growth as air temperatures rise. In contrast, younger cohorts of both *Taxodium* species showed unexpected increases in growth rates through time. Also notable was the fact that while there were marked differences in growth rates of young individuals with *P. elliottii* having higher growth rates than *Taxodium* species, all species converged at similar growth rates once they surpassed 70 years of age. This suggests that despite early‐life advantages of faster‐growing species like *P. elliottii* , long‐term resource limitations and structural constraints lead all species to similar growth rates. Ecologically, this implies that initial competitive hierarchies among taxa may diminish over time, promoting coexistence and stabilizing forest structure. It also highlights how life‐history strategies shift, from accelerated juvenile growth to resource‐conservative growth in maturity, affecting succession dynamics and community composition at the landscape scale.

In synthesis, south Florida's dominant tree species have different ways to acclimate to changes in climate and environmental conditions. Growth rates in all species are driven by water depth with little influence of climatic variables (i.e., precipitation, temperature, and VPD). Thus, changes in hydrologic conditions greatly impact the growth of our focal species in south Florida landscapes. Conversely, iWUE increased in all species and was driven by temperature, VPD, and *C*_*a*_, whereas precipitation and water depth had differential effects on species' iWUE. Finally, we observed that growth rates decreased over time in *P. elliottii* , but we did not find changes in growth rates over time in either *Taxodium* species.

These results suggest that hydrologic variability, more than direct temperature, rainfall, or VPD shifts, ultimately governs how these three species grow in south Florida. All three species track changes in water depth and their microsite positions when water levels rise or fall. These changing water levels alter oxygen availability in the root zone, nutrient fluxes, and competitive dynamics, all of which immediately translate into faster or slower radial growth. In contrast, climatic factors (precipitation, temperature, VPD) exert only a weak direct control on growth rates, though they do drive changes in iWUE.

Because *P. elliottii* growth rates have declined over recent decades (even as its iWUE rose), it appears more vulnerable to changes in climate and hydrology, likely a result of interacting hydrologic stress (e.g., deeper or more prolonged inundation) and rising evaporative demand. In contrast, both *Taxodium* species maintained stable growth rates over time despite similar increases in iWUE. In other words, they are buffering their carbon–water economy: by boosting iWUE under hotter, drier atmospheric conditions, they avoid having to drastically reduce stem growth when floods or droughts occur.

Between the two cypress species, the dwarf ecotype of *T. ascendens* is particularly well suited to withstand future climate and hydrological variability. Its characteristic low, stunted form reflects a long‐term strategy of slow growth under extremely high or fluctuating water tables. Because its rings, and thus its carbon allocation, are already tuned to alternating periods of inundation and drought, any further shifts in hydrology (e.g., more extreme dry downs or deeper floods) are less likely to push it beyond critical thresholds. By contrast, a faster growing species like *P. elliottii* won't be able to sustain high growth when water conditions fluctuate dramatically; its declining growth trend suggests that it may be less able to acclimate as water regimes continue to change.

## Author Contributions

**Manuel Bernal‐Escobar:** conceptualization (equal), data curation (lead), formal analysis (lead), funding acquisition (equal), investigation (equal), methodology (lead), project administration (equal), writing – original draft (lead), writing – review and editing (equal). **Courtney L. Angelo:** conceptualization (equal), data curation (supporting), formal analysis (supporting), funding acquisition (equal), investigation (equal), writing – original draft (supporting), writing – review and editing (equal). **Kenneth J. Feeley:** conceptualization (equal), data curation (supporting), formal analysis (equal), funding acquisition (equal), investigation (supporting), writing – original draft (supporting), writing – review and editing (equal).

## Conflicts of Interest

The authors declare no conflicts of interest.

## Supporting information


**Data S1:** ece373253‐sup‐0001‐DataS1.docx.

## Data Availability

The data and code that support the findings of this study are openly available in Zenodo at https://doi.org/10.5281/zenodo.15635388 (Bernal‐Escobar 2025).
