# Supplementary material for: Climate and Hydrology Shape the Growth and Water Use Efficiency in South Florida's (USA) Pine and Cypress Forests
Source: Ecol Evol. 2026 Apr 5;16(4):e73253. doi: 10.1002/ece3.73253 (PMC13052257; doi:10.1002/ece3.73253)
Supplement: Supplementary file 1 — Data S1: ece373253‐sup‐0001‐DataS1.docx. [file ECE3-16-e73253-s001.docx]

## **Supplementary information**

Supp. Table 1: Summary statistics for dated tree-ring width series included in the chronologies in Big Cypress National Preserve, Florida. MS: mean sensitivity. r_xy_: series intercorrelation.

| **Species** | ***T. ascendens*** | ***T. distichum*** | ***P. elliottii*** |
| --- | --- | --- | --- |
| Number of trees | 22 | 26 | 25 |
| Number of dated series | 43 | 49 | 52 |
| Master series (years) | 289 | 219 | 152 |
| Total rings in all series | 7484 | 4352 | 3525 |
| Total dated rings checked | 7470 | 4337 | 3523 |
| Series intercorrelation (r_xy_) | 0.427 | 0.407 | 0.457 |
| Average mean sensitivity (MS) | 0.719 | 0.670 | 0.568 |
| Mean length of series | 174.0 | 88.8 | 67.8 |


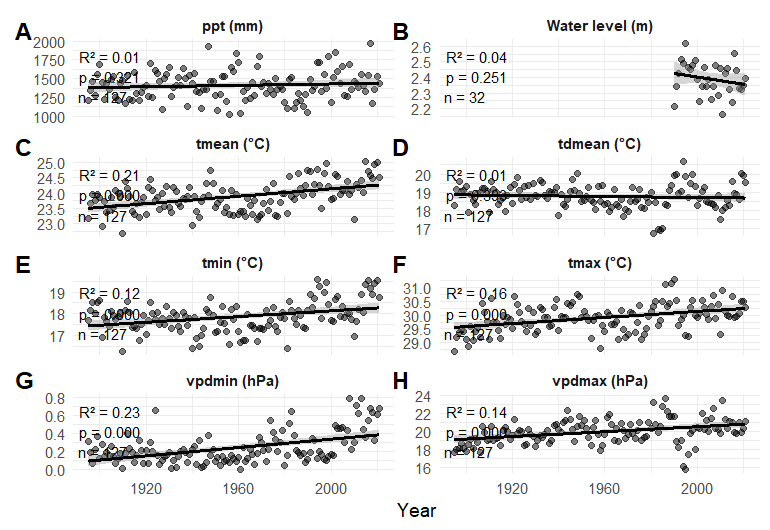


Supp. Figure 1: Climate time series and fit linear models. A. Precipitation. B. Water level. C. Mean temperature. D. Tdmean (Mean temperature at dew point). E. Minimum temperature. F. Maximum temperature. G. minimum VPD. H. Maximum VPD. R^2^, p=p-value, n= sample size.

*
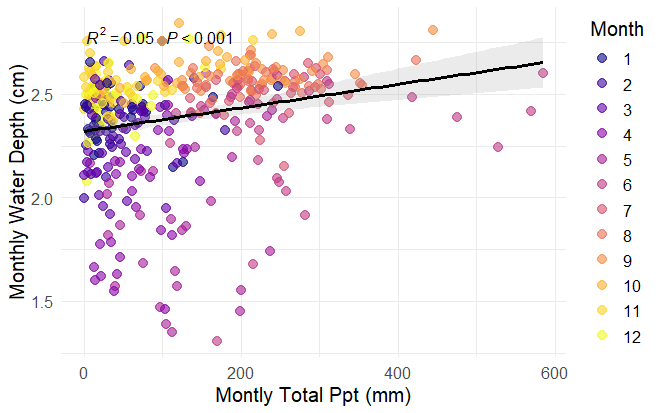
*

Supp. Figure 2: Linear regression between monthly total precipitation and monthly water depth.


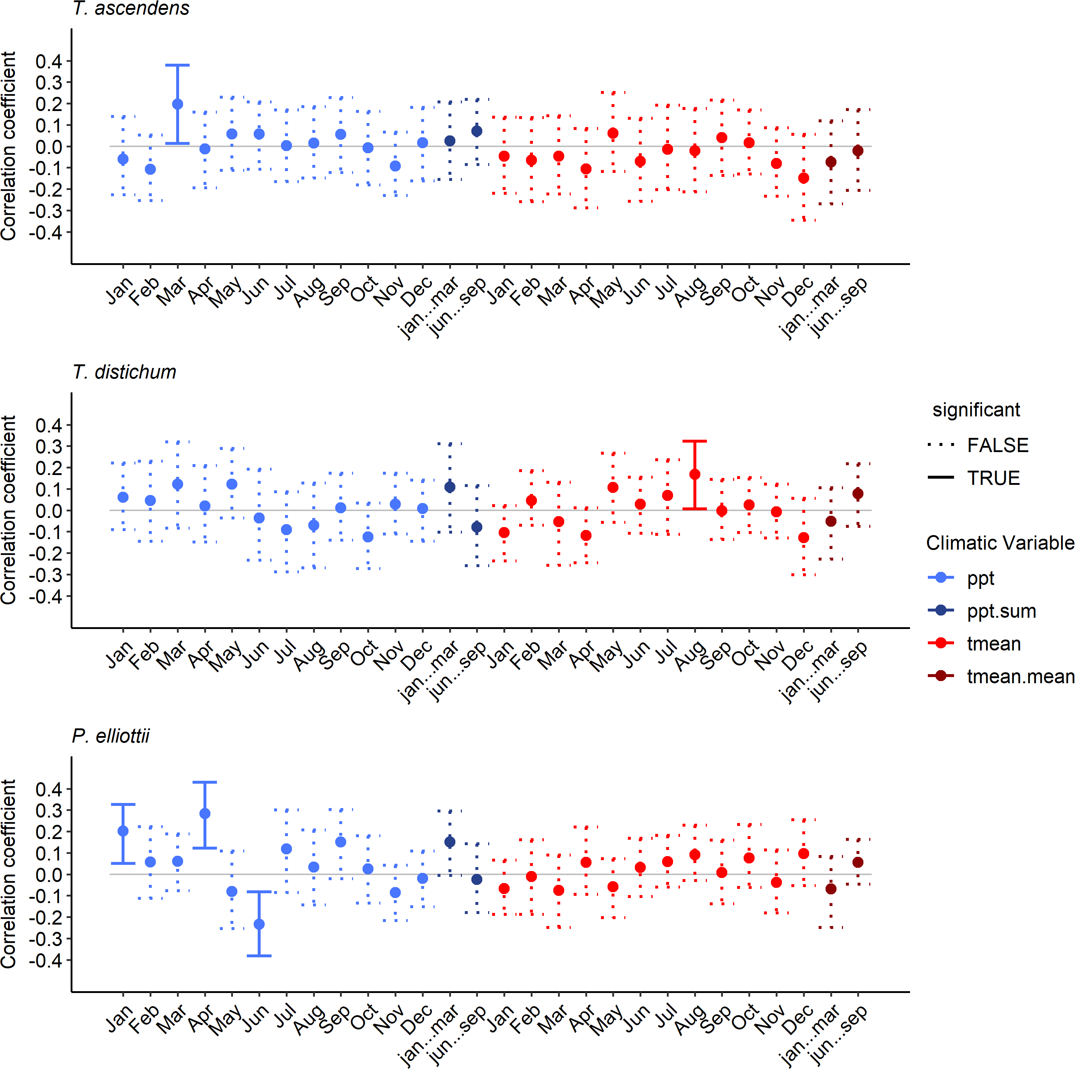


Supp. Figure 3: Pearson correlation values between T. ascendens, T. distichum, and P. elliottii residual chronologies and previous year monthly mean temperature and precipitation (PRISM Climate Group and Oregon State University 2023).


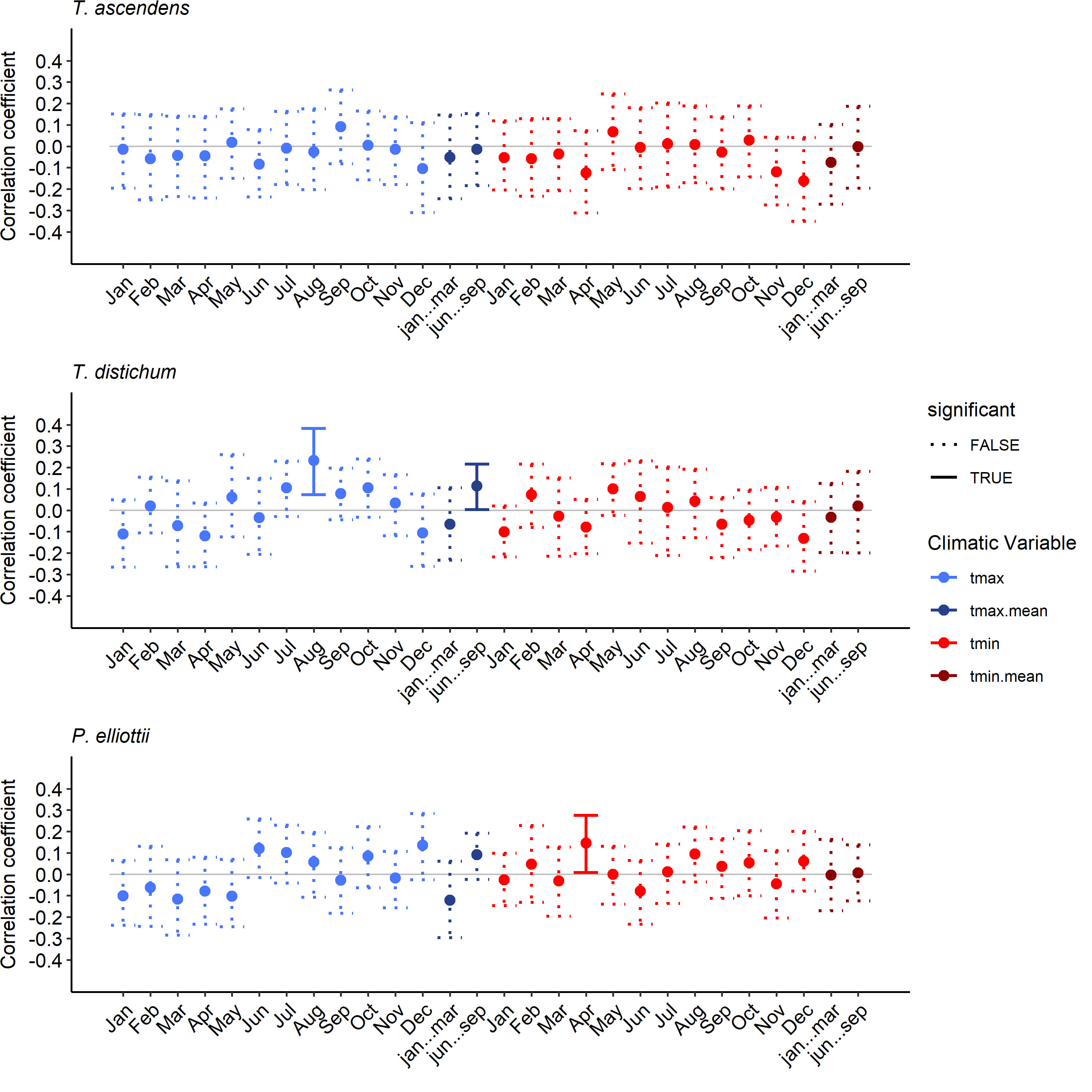


Supp. Figure 4: Pearson correlation values between T. ascendens, T. distichum, and P. elliottii residual chronologies and previous year monthly maximum and minimum temperature (PRISM Climate Group and Oregon State University 2023).


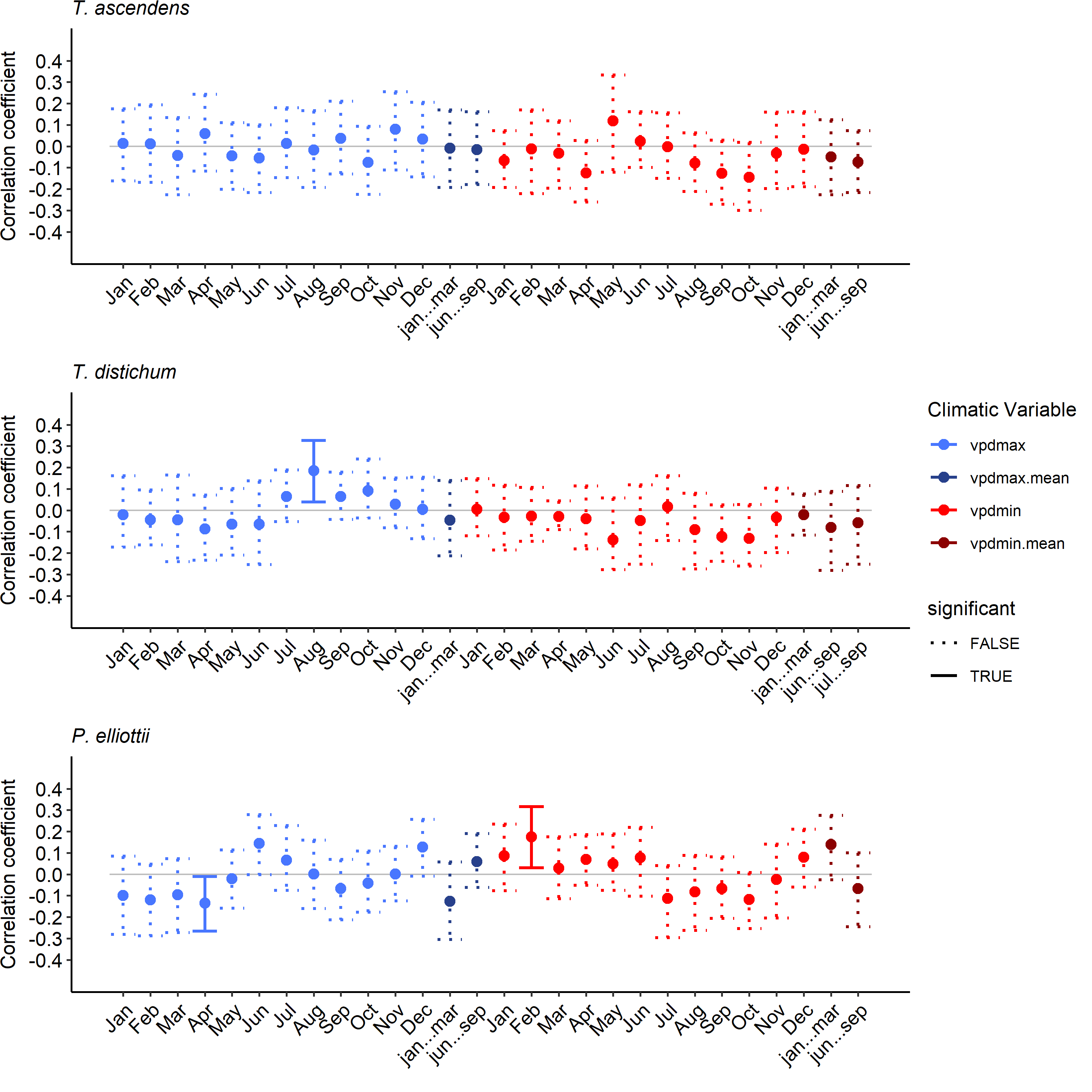


Supp. Figure 5: Pearson correlation values between P. elliottii, T. ascendens, and T. distichum residual chronologies and previous year monthly maximum and minimum vapor pressure deficit (VPD)(PRISM Climate Group and Oregon State University 2023).


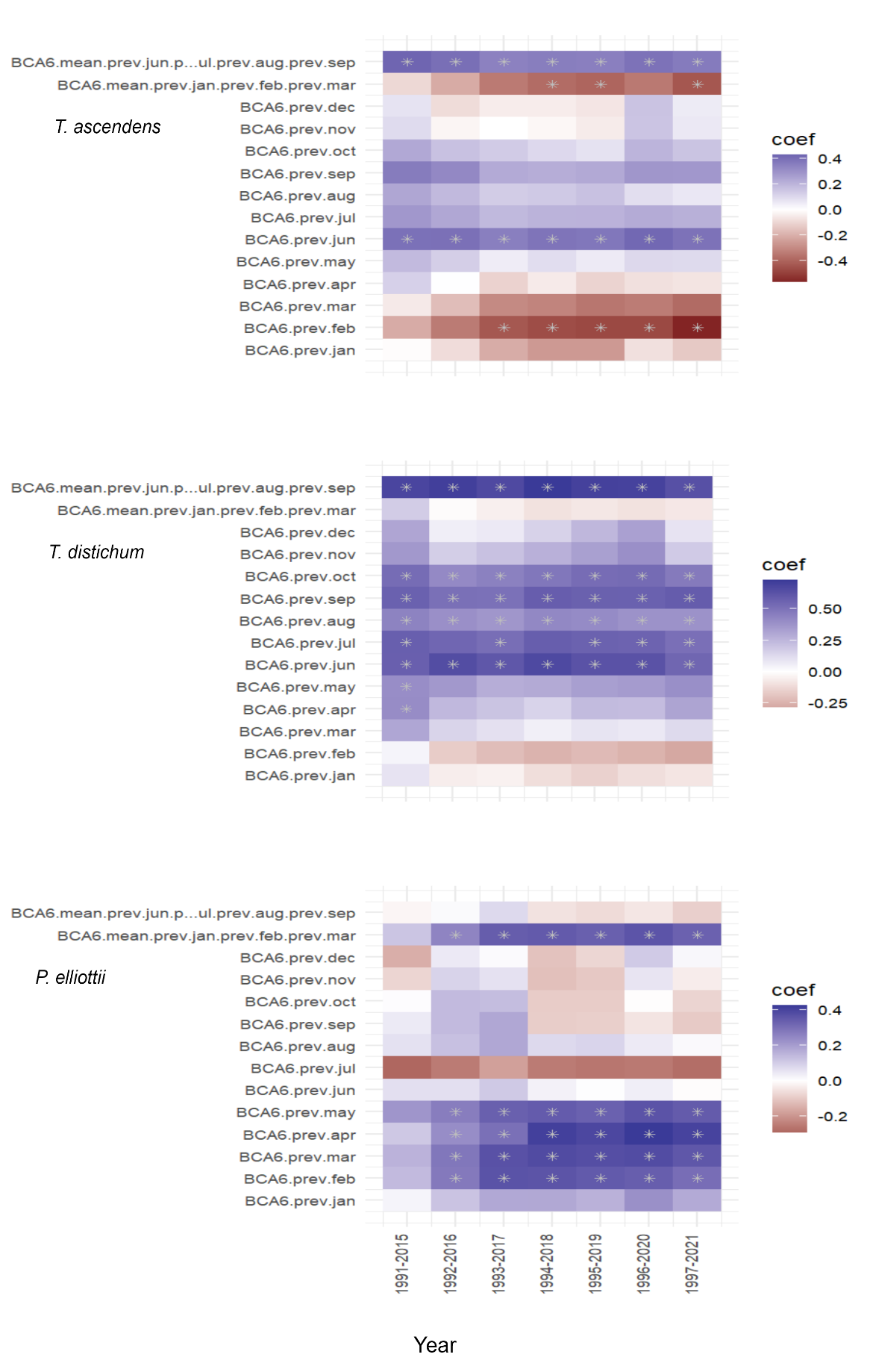


Supp. Figure 6: Pearson correlation values over time between T. ascendens, T. distichum, and P. elliottii RWI chronologies and previous year water level monthly data from station BCA6 from 1990-2021

.


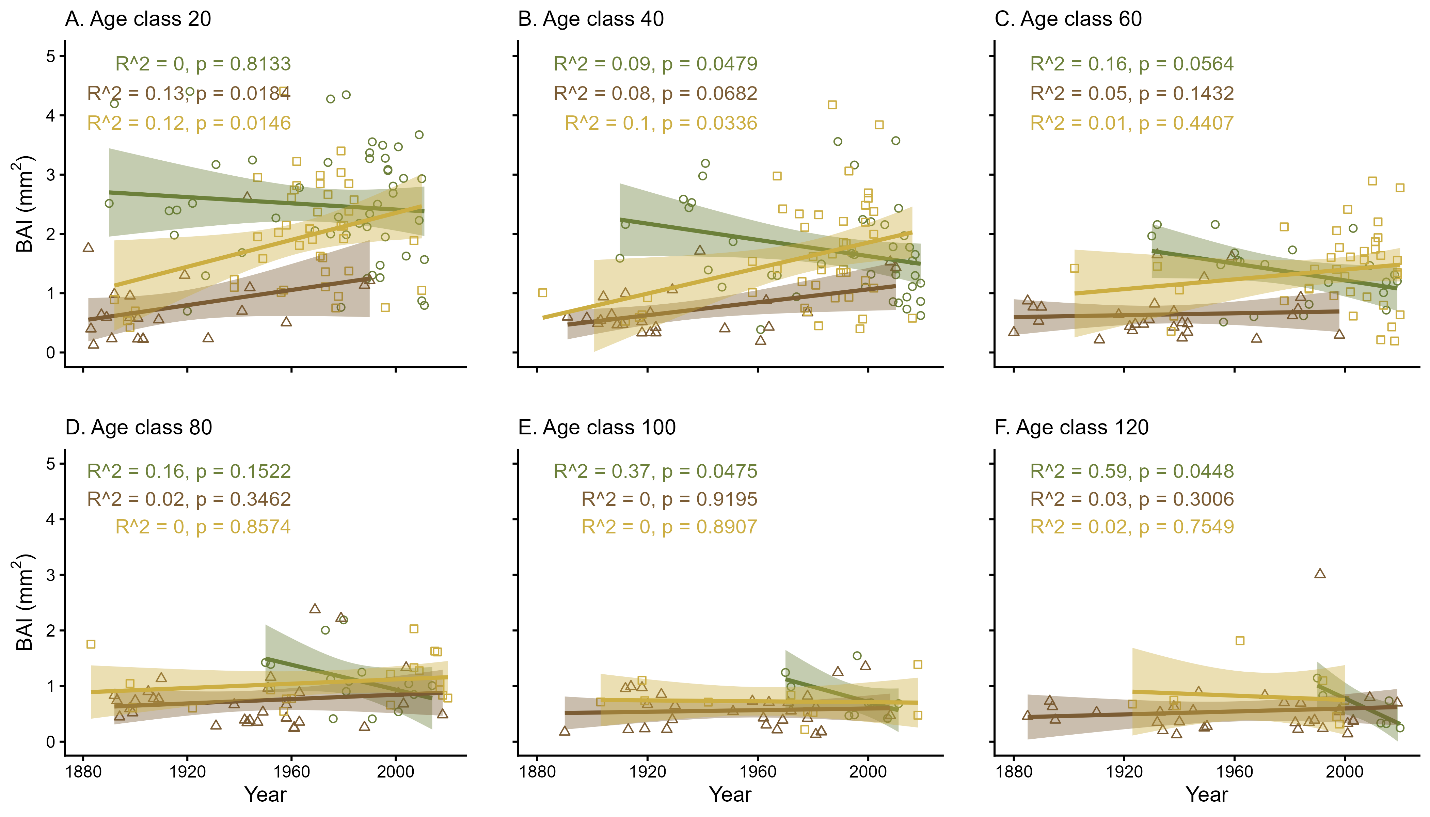


Supp. Figure 7: Age Class Analysis for: A. Age class 20, B. Age class 40, C. Age class 60. D. Age class 80. E. Age class 100. F. Age class 120. Y axis: Basal Area Index (mm^2^), X axis time in years. T. ascendens in brown, T. distichum in yellow, and P. elliottii in green.


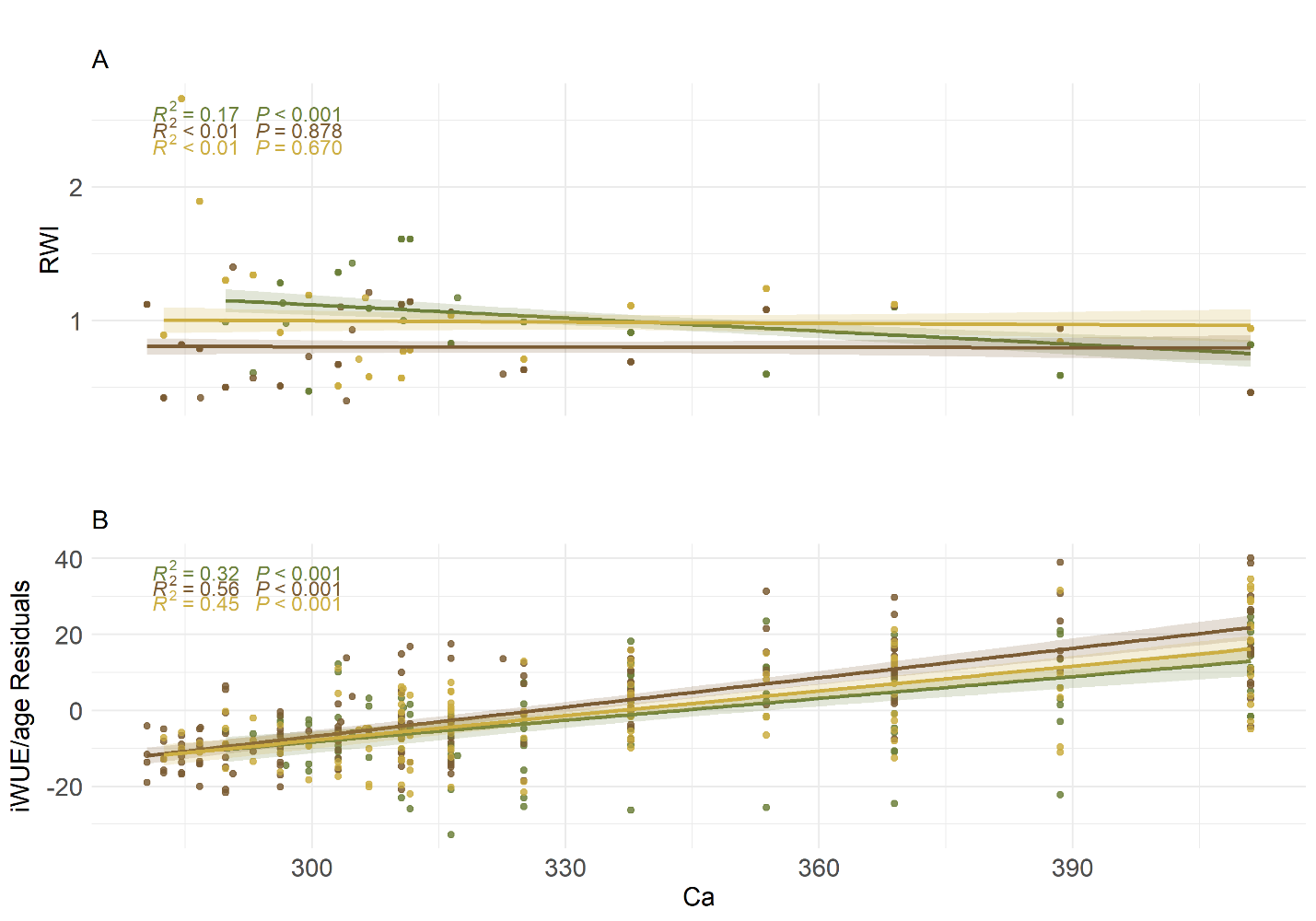


Supp. Figure 8: Linear regression between A. Ring Width Index (RWI) and atmospheric carbon concentration (C_a_). B. iWUE and C_a_. T. ascendens in brown, T. distichum in yellow, and P. elliottii in green.
